# Concurrent Outbreaks of Cholera and Peripheral Neuropathy Associated with High Mortality among Persons Internally Displaced by a Volcanic Eruption

**DOI:** 10.1371/journal.pone.0072566

**Published:** 2013-09-02

**Authors:** Alexander Rosewell, Geoff Clark, Paul Mabong, Berry Ropa, Enoch Posanai, Nicola W. Y. Man, Samir R. Dutta, Wasa Wickramasinghe, Lixia Qi, Jack C. Ng, Glen Mola, Anthony B. Zwi, C. Raina MacIntyre

**Affiliations:** 1 World Health Organization, Port Moresby, National Capital District (NCD), Papua New Guinea; 2 School of Public Health and Community Medicine, The University of New South Wales, New South Wales, Sydney, NSW, Australia; 3 Provincial Health Office, Madang, Madang Province, Papua New Guinea; 4 National Department of Health, Port Moresby, NCD, Papua New Guinea; 5 Pathology Department, Port Moresby General Hospital, Port Moresby, NCD, Papua New Guinea; 6 The University of Queensland, National Research Centre for Environmental Toxicology, Coopers Plains, Queensland, Australia; 7 University of Papua New Guinea, Port Moresby, NCD, Papua New Guinea; Wadsworth Center, United States of America

## Abstract

**Background:**

In October 2004, Manam Island volcano in Papua New Guinea erupted, causing over 10 000 villagers to flee to internally displaced person (IDP) camps, including 550 from Dugulaba village. Following violence over land access in March 2010, the IDPs fled the camps, and four months later concurrent outbreaks of acute watery diarrhea and unusual neurological complaints were reported in this population.

**Materials and Methods:**

A retrospective case-control study was conducted to identify the risk factors for peripheral neuropathy. Rectal swabs were collected from cases of acute watery diarrhea. Hair and serum metals and metalloids were analyzed by Inductively Coupled Plasma-Mass Spectrometry (ICP-MS).

**Results:**

There were 17 deaths among the 550 village inhabitants during the outbreak period at a crude mortality rate 21-fold that of a humanitarian crisis. *Vibrio cholerae* O1 El Tor Ogawa was confirmed among the population. Access to community-level rehydration was crucial to mortality. Peripheral neuropathy was diagnosed among cases with neurological symptoms. A balanced diet was significantly protective against neuropathy. A dose-response relationship was seen between peripheral neuropathy and a decreasing number of micronutrient- rich foods in the diet. Deficiencies in copper, iron, selenium and zinc were identified among the cases of peripheral neuropathy.

**Conclusions:**

Cholera likely caused the mostly preventable excess mortality. Peripheral neuropathy was not caused by cholera, but cholera may worsen existing nutritional deficiencies. The peripheral neuropathy was likely caused by complex micronutrient deficiencies linked to non-diversified diets that potentially increased the vulnerability of this population, however a new zinc-associated neuropathy could not be ruled out. Reoccurrence can be prevented by addressing the root cause of displacement and ensuring access to arable land and timely resettlement.

## Introduction

Excess cholera mortality can be caused by a variety of factors, including delayed reporting, lack of preparedness, host-factor vulnerability, such as malnutrition, and health system access [1]. The goal for mortality-reduction activities during cholera outbreaks is a case-fatality ratio below 1%, achieved through rapid community-level access to rehydration and effective referral systems [2]. The cholera outbreak in Papua New Guinea has been associated with widespread morbidity and mortality, with limited health system access, and weak outbreak identification and response capacity contributing to excess mortality.

Neurological manifestations are important contributors to morbidity and mortality during the acute phase of humanitarian emergencies as well as in the subsequent months and years [3]. Outbreaks of optic and peripheral neuropathy, for which the identification of a definitive cause is challenging, have been reported in association with dietary inadequacies [4], [5]. Despite numerous reports of both cholera [6] and peripheral neuropathy [3] among internally displaced persons (IDPs), the epidemiological features of their concurrent outbreaks and their impact on mortality have not been previously described.

Papua New Guinea is prone to natural disasters and population displacement. In October 2004, Manam Island volcano erupted, causing over 10 000 villagers to flee to IDP camps [7]. The IDP population was initially provided food rations because numerous sections of fishing reefs were destroyed and there was limited or no access to crops or to land for food production. Following the burning of houses and killings between IDPs and local residents over land access in and around the IDP camps on the mainland in March 2010, IDPs fled to their home village from the IDP camps. In early July 2010, a Manam Island villager reported an outbreak of acute watery diarrhoea and neurological symptoms associated with unusual mortality to provincial health authorities.

We aimed to determine the cause and extent of concurrent outbreaks of acute watery diarrhoea and peripheral neuropathy with unusual mortality in the setting of a natural disaster, and to implement prevention and control measures.

## Materials and Methods

### Setting and population

Cholera transmission had been identified in Madang Province since September 2009. After 16 community deaths had occurred between 17 and 28 June 2010, a villager reported the outbreak to the provincial cholera coordinator. An investigation was conducted at the Potsdam Care Centre, located on the north coast of the Papua New Guinea mainland in Bogia District, Madang Province. The centre was one of several established by authorities in 2004 in response to population displacement that followed a volcanic eruption on the island. The displaced person population along the Bogia coast was estimated to be approximately 10 000, of which 550 lived in Potsdam. We investigated two health events in Potsdam: 1) the outbreak of acute watery diarrhoea and associated deaths, and 2) unusual neurological complaints reported among the Potsdam Care Centre residents.

### Data collection

A standardized questionnaire was used to gather demographic, clinical and environmental information from health-care workers and family members of those who had died since the beginning of the outbreak. A suspected cholera case was defined as acute watery diarrhoea or vomiting in a care centre resident since 17 June 2010. A confirmed cholera case was defined as a suspected case, with a positive stool culture. A suspected cholera death was defined as a person with a case of suspected cholera who died within seven days of the onset illness. An active case finding (house to house) was conducted to identify villagers reporting dysesthesia or other neurological complaints, inviting them to a central location for assessment by a clinician the following day. A clinician obtained neurological histories and used clinical methods to assess motor strength, sensitivity to light touch and pinpricks, cerebellar function and gait, and deep-tendon reflexes on people reporting the recent onset (within three months) of unusual symptoms. A case of peripheral neuropathy was defined as a resident presenting to the assessment site with numbness and edema, or diminished proprioception and pinprick, or light touch perception, or decreased strength, or paraesthesia, or deep tendon hyporeflexia of the lower limbs.

### Analytical epidemiology

A retrospective case-control study was conducted to identify the risk factors associated with peripheral neuropathy, using neighborhood controls selected in a ratio of 5:1. Demographic, clinical, laboratory and behavioral data, as well as a 90-day food history, were collected using a standardized questionnaire. Recent history of any symptoms compatible with neuropathy served as the exclusion criterion for the selection of community controls. We developed a food-diversity score variable for each participant based on the number of distinct micronutrient-rich food items consumed during the investigation period. Fish (either canned or reef), shellfish, bananas, coconuts and greens were included due to their micronutrient content, while instant noodles, tea and oil were excluded.

### Data management and statistical analysis

Data was entered into Microsoft Excel for cleaning and coding. As appropriate for the small sample size in this study, Fisher’s exact test and the exact 95% confidence interval of the odds ratio (OR) were used for the univariate analyses on association of potential risk or protective factors with peripheral neuropathy. We used the χ2 test for linear trend to assess the statistical significance of food diversity. Exact binary logistic regression was performed to test association of risk or protective factors with peripheral neuropathy. Where there is complete separation of data, median unbiased estimates of OR and its 95% CI was used [8], [9]. All statistical analyses were performed using Stata version 10 (Stata Corp., College Station, TX, USA).

### Microbiology

Rectal swabs were collected from anyone reporting recent diarrheal illness at the care centre and nearest health centre. Swabs were inoculated into Cary-Blair media [10] then transported to the national reference laboratory, where they were plated directly onto thiosulfate citrate bile salt sucrose (TCBS) agar (Oxoid Ltd. U.K.). The swabs were also inoculated into alkaline peptone water (pH 6.8) for enrichment of vibrios and incubated for 6 to 8 hours then plated onto thiosulfate citrate bile salt sucrose (TCBS) agar. These were incubated at 370C for 18 to 24 hours. Typical yellow colonies of V. cholerae were biochemically tested and confirmed by agglutination with polyvalent O1 and monovalent Ogawa and Inaba antisera (Remel Europe Ltd. U.K.). Non-agglutinating strains were tested with antiserum to V. cholerae O139 strain.

### Elemental Analysis

Whole blood samples were collected from all cases of peripheral neuropathy and placed in plastic blood tubes with EDTA and immediately stored at 4oC prior to freezing at –70oC for shipment to external reference laboratories. Hair samples were collected from four cases of peripheral neuropathy by removing hair from the scalp with a standard scissors and storing in plastic bags for subsequent transport. Water samples of approximately 2 liters were collected in sterile glass bottles from the drinking-water point in the village on the island, stored at 4oC and transported to the laboratory for heavy-metals testing.

Serum metals and metalloids were analyzed by ICP-MS (Agilent 7500CS, Agilent Technologies, Australia) after dilution in an alkaline solution and addition of internal standards [11]. Briefly, 0.25 mL of serum was diluted in 0.5 mL alkaline solution, 4.25 mL of Milli-Q deionized water (Millipore, Bedford, Massachusetts, United States of America) and 0.1 mL of internal standards prior to ICP-MS analysis at the National Research Centre for Environmental Toxicology, Australia. Reagent blanks, spiked samples and certified reference material (CRM) blood were done similarly. The recovery from spiked samples ranged from 90%–108% and the results from the blood CRM were within the acceptable certified values. Blood specimen testing for thiamine levels was conducted through Queensland University, Australia. Thiamine levels were determined by cobas and microplate assay of erythrocyte transketolase activation coefficient performed in lysates.

Water samples collected from village sources were tested for heavy metals by adding 0.1 mL Internal Standards (I.S.) to 5 mL water samples and 5 mL Certified Reference Material (TM28.3 Lot 1107 from Environmental Canada) for ICP-MS analysis. The internal standards contained appropriate elements at 500 μg/L in 10% HNO3 and 5% HCl. Multi-Element Calibration standard-2A (Agilent Technologies) was used to prepare the external standards range from 0 μg/L –200 μg/L.

Hair samples were washed based on an established method [12]. After drying, the hair was weighed accurately into a 10 mL natural colored plastic tube and digested in ultra pure nitric acid (1 mL) at 70°C over night. The digest was then diluted to 10 mL. Aliquots of this solution (1 mL) were diluted to 4 mL of Milli-Q deionized water and 0.1 mL of internal standards followed by ICP-MS analysis. Reagent blanks and Certified Reference Material–hair (NCS DC73347) were prepared and analyzed in the same manner. The recovery from spiked samples ranged from 94%–103% and the results from the CRM-hair were within the acceptable certified values.

### Control measures

An oral rehydration point was established in the community for early treatment and referral of diarrhoeal cases. Meetings were held with district health authorities to reactivate the district cholera task force and to strengthen early warning and response mechanisms. Food education, including information about food preparation and vitamin antagonists such as betel nut, tea and coffee, was conducted to ensure maximum nutritional benefit from available foods, and a meeting was held with the district administration to advocate for a more diversified food ration.

### Ethics statement

All subjects provided verbal informed consent to participate in the investigations. As this was a formal outbreak investigation conducted in line with national and World Health Organization (WHO) guidance, prior ethical approval was not required. However, the Western Pacific Regional Office of WHO’s Ethics Review Committee reviewed the procedures that were followed during the investigation, based on the information provided by the investigation team, and agreed that the investigation was in compliance with key ethical principles. All investigation participants and data collected therein were treated following the ethical principles outlined in the Helsinki Declaration. Specifically, the IDPs under investigation were given the choice to refuse to participate, relevant samples were collected ensuring complete privacy, and the investigation findings were fed back to the community. Data confidentiality was maintained at all stages of data management from collection to storage. Informed verbal consent was not documented, however the IDPs understood that their participation was voluntary and interviews were conducted in the presence of the village leader.

## Results

### Descriptive epidemiology

We identified 17 deaths among the 550 village inhabitants over a 15-day period, giving a crude mortality rate of 21/10 000 population per day. Sixteen deaths occurred prior to investigation with one occurring during the investigation. Fifteen (88%) deaths were associated with diarrhoea, one (6%) with placenta expulsion during a birth in the bushes, and one (6%) with collapse of an unknown cause. Fifteen deaths (88%) were of people aged 20 years or over, of which five were aged 25 to 34 years (average age: 42 years; range 6–70 years). The majority of deaths occurred at home (59%), with fewer during boat transport to the health facility (24%) or once admitted to a health facility (17%). No oral rehydration point was established in the community prior to the intervention. Cost was the main reason for people not accessing health care, with the required boat hire costing approximately US$ 40. The average time from symptom onset to death among fatal cases was one day. The only death the response team arrived in time to investigate was a 26-year-old female who demonstrated symptoms of pulmonary edema at time of death, five to six days after an episode of acute watery diarrhoea.

### Microbiology

The last fatal case (26 years old) was negative for *Vibrio cholerae* and positive for *Aeromonas caviae*. One resident was confirmed with *Vibrio cholerae* O1 El Tor Ogawa (the nationally circulating strain).

### Clinical characteristics of peripheral neuropathy

Mixed neurological signs and symptoms were reported among the 13 persons identified through active case finding and were physically assessed by a clinician. Decreased strength and numbness in the lower limbs were common, as well as diminished proprioception and pinprick or light touch perception of lower limbs. Bilateral decrease in auditory acuity was reported in one person that met the case definition, who could only be tested at the gross level, but responded to audible sounds in both ears. There was no evidence of abnormalities in postural stability sway speed, spastic paraparesis or signs of optic neuropathy. One case of peripheral neuropathy reported experiencing acute watery diarrhoea during the acute outbreak or within the previous three months, however laboratory investigations were not conducted at the time of illness.

### Analytical epidemiology

Eight people met the case definition for peripheral neuropathy, of which seven, along with 36 neighborhood controls, participated in the case-control study. One case of peripheral neuropathy reported experiencing acute watery diarrhoea during the acute outbreak or within the previous three months. There was no difference between the two study populations in terms of age or sex (see Table 1). In the absence of meats, the predominant source of protein for this coastal population was fish. A diverse diet in the last 90 days was more likely to be reported by controls on univariate analysis (see Table 2). A diverse diet was the only factor associated with protection on exact binary logistic regression analysis (aOR 0.12; 95% CI 0.00, 0.95 p<0.05) (see Table 2). There was a significant trend towards greater protection from peripheral neuropathy with increased dietary diversification (see Table 3).

**Table 1:** Demographic characteristics of study subjects, Papua New Guinea, 2010.

Demographic characteristic		Cases		Controls	
		n = 7	(%)	n = 36	(%)
Sex	Male	5	(71)	16	(44)
Age group	20–29	3	(43)	12	(33)
	30–39	3	(43)	10	(28)
	40–49	0	(0)	12	(33)
	50–59	1	(14)	2	(6)

**Table 2:** Risk factors associated with peripheral neuropathy, Papua New Guinea, 2010.

Exposure	Cases		Controls		Crude odds ratio	Confidence interval	Adjusted odds ratio	Confidence interval
	n = 7	(%)	n = 36	(%)	(cOR)	(95%CI)	(aOR)	(95%CI)
**Acute watery diarrhoea last 30 days**	1	(14)	7	(19)	0.70	(0.01, 7.43)	-	-
**Acute watery diarrhoea last 12 months**	3	(43)	11	(31)	1.68	(0.21, 11.90)	-	-
**Acute respiratory illness last 30 days**	2	(29)	8	(22)	1.39	(0.11, 10.73)	-	-
**Acute respiratory illness last 12 months**	5	(71)	9	(25)	7.09	(0.96, 86.76)	-	-
**Greens 90 days**	6	(86)	31	(86)	0.97	(0.08, 53.30)	-	-
**Fish 90 days**	5	(71)	35	(97)	0.08	(<0.01, 1.77)	0.30	(<0.01, 23.55)
**Oil 90 days**	7	(100)	35	(97)	0.19†	(0.01, +∞)	-	-
**Bananas 90 days**	7	(100)	34	(94)	0.47†	(0.03, +∞)	-	-
**Coconut 90 days**	6	(86)	36	(100)	0.19†	(<0.01, 7.58)	-	-
**Instant noodles 90 days**	7	(100)	34	(94)	0.47†	(0.04, +∞)	-	-
**Tea 90 days**	7	(100)	34	(94)	0.47†	(0.04, +∞)	-	-
**Large fish (fresh) 90 days**	2	(29)	24	(67)	0.21	(0.02, 1.50)	-	-
**Shellfish 90 days**	1	(14)	11	(31)	0.39	(0.01, 3.82)	-	-
**Attend funeral last 30 days**	6	(86)	29	(81)	1.44	(0.14, 76.01)	-	-
**Trauma 30 days**	7	(100)	31	(86)	1.41†	(0.17, +∞)	-	-
**Travel to town 90 days**	1	(14)	16	(44)	0.22	(<0.01, 2.06)	0.15†	(<0.01, 1.28)
**Returned to island in recent crisis**	7	(100)	28	(78)	2.54†	(0.33, +∞)	-	-
**Access to garden 90 days**	0	(0)	10	(28)	0.29†	(<0.01, 2.22)	-	-
**Diverse diet** **	4	(57)	33	(92)	0.12	(0.01, 1.30)	0.12† *	(<0.01, 0.94)

* P-value <0.05.

† unbiased estimates.

** those with >3 foods against those with ≤3 foods in their diet.

**Table 3:** Diversity of diet among cases of peripheral neuropathy, Papua New Guinea, 2010.

Dietary diversity	Cases		Controls		Odds ratio	Confidence interval	Test for trend of OR	Test of homogeneity
	n = 7	(%)	n = 36	(%)	(OR)	(95%CI)		
≤3 foods	3	(43)	3	(8)	1.00	–	–	
4 foods	4	(57)	24	(67)	0.18	(0.02, 1.81)	0.17	
5 foods	0	(0)	9	(25)	0.12	(0.00, 1.35)	0.09	0.02

### Elemental Analysis

Analysis of seven blood samples collected from cases of peripheral neuropathy identified deficiencies in zinc in all, as well as copper, iron, selenium (see Table 4), with no vitamin B1 (thiamine pyrophosphate) deficiencies identified. There was no evidence of heavy-metal contamination or toxicity from the analysis of blood, hair or water samples.

**Table 4:** Serum analysis (µg/L) of peripheral neuropathy cases, Papua New Guinea, 2010.

	Vitamin B1	Cr	Fe	Cu	Zn	As	Se	Ag	Cd	Hg	Tl	Pb
Normal range	28–85	0.3– 1.4 [29]	560– 2128 [29]	756– 1386 [29]	654– 1177 [29]	0– 9.8 [29] *	59– 107 [29]	0.1– 0.5 [30] *	0– 10.1 [29] *	0– 10 [29] *	0– 1.0 [30] *	0– 100 [30] *
Case 1	-	0.4	**395.4**	1110.6	**136.1**	UDL	77.5	5.3	8.3	4.2	3.7	2.3
Case 2	40	0.5	734.6	847.4	**382.2**	10.758	**38.5**	2.5	8.9	3.5	3.6	13.6
Case 3	50	0.7	822.2	**289.2**	**202.2**	UDL	**31.8**	2.6	10.2	3.7	4.0	7.0
Case 4	55	1.3	**430.2**	**459.4**	**81.72**	UDL	**24.0**	2.3	9.5	3.3	3.8	6.7
Case 5	40	0.5	1270.6	**256.4**	**147.3**	UDL	71.8	2.2	10.0	3.3	4.0	10.6
Case 6	60	0.8	**190.3**	**609.6**	UDL	UDL	**21.8**	2.0	8.1	3.3	3.4	UDL
Case 7	-	0.4	**76.0**	**300.4**	**186.7**	UDL	61.1	1.9	9.2	3.0	3.8	5.9

* normal range in whole blood.

UDL  =  under detection limit.

Below normal range in bold [29], [30].

## Discussion

Most excess mortality among IDPs is entirely preventable [13]. Papua New Guinea is estimated to have a high risk of adult mortality. Gender-specific probabilities of dying between 15 and 60 years per 1000 population are 283 (249–320) for females and 380 (343–422) for males [14]. However these events increased the risk of mortality to extreme levels above the background rate. Six years after this population was initially displaced, the crude mortality rate during this outbreak exceeded by 21-fold the threshold for a humanitarian emergency [15]. Fast outbreak detection, reporting and response are crucial in preventing outbreak-associated morbidity and mortality [16]. For diarrheal outbreaks, effective response includes the use of low osmolarity oral rehydration salts (ORS) and zinc [17]. Despite previous cholera transmission in this district, such systems were weak and 16 deaths occurred in this small community before the outbreak was reported, limiting the opportunity to save lives. Zinc supplementation is associated with substantial reductions in the rates of diarrhoea and pneumonia, the two leading causes of death in developing countries [18]. Despite zinc being part of the national policy for diarrheal disease management since 2005, it was not available before the outbreak nor was it widely available during the outbreak. Further work is required to institutionalize zinc for diarrheal disease management. Access to care was crucial to the high mortality, reflected by the 59% of deaths that occurred in the home, with another 24% in transit to the health facility at a prohibitive cost to many villagers. Once an oral rehydration point was established at the community level, there were no further fatal cases. These findings highlight the health system access challenges these communities face during outbreaks and more generally, but also highlight the effectiveness of access to ORS at the community level.

Malnutrition is arguably the major contributor to global morbidity and mortality, and renders displaced populations more vulnerable to the health impacts of acute outbreaks of infectious diseases. Malnutrition and its interconnectedness with communicable diseases including diarrhoea [19], explains much of the excess mortality seen among displaced populations, with key micronutrients playing a role in increasing vulnerability to infection and death. Copper, iron, selenium and zinc are all known to confer function in both innate and adaptive immune systems, with copper also impacting intestinal immune barriers [20]. A history of prior cholera infection has been identified as a risk factor for outbreaks of thiamine deficiency among prisoners [21], and food diversity has recently been shown to be protective against cholera in the ongoing outbreak in Haiti [22]. In our study, it would appear these outbreaks were concurrent and that symptomatic cholera infection did not cause the micronutrient deficiencies among the cases of peripheral neuropathy, as the majority (88%) of cases did not report an episode of diarrhoea in the previous three months. However, underlying malnutrition would have exacerbated the effects of the cholera outbreak. The combination of deficiencies seen in this population (copper, iron, selenium and zinc) may have increased the susceptibility to cholera infection and vulnerability to cholera-associated mortality.

This is the first time that the concurrent occurrence of cholera and peripheral neuropathy has been reported in a displaced person population. While the two diseases do not appear to be directly related, the presence of underlying malnutrition may increase susceptibility to cholera, and conversely, acute cholera may unmask and precipitate subclinical micronutrient deficiencies. Conditions such as neuropathy may become more prominent in a setting of acute outbreaks of infectious diseases. Large-scale outbreaks of peripheral neuropathy have been associated with food shortages and low intakes of animal fat and protein [23]. Similar to previously investigated outbreaks [4], [5], overt malnutrition was not present in this population, but multiple micronutrient deficiencies were identified. Of the malnutrition-related neurological disorders, peripheral neuropathy has been associated with several micronutrient deficiencies, including copper [24], chromium, pyridoxine (B6) [25] and cobalamin (B12) and thiamine (B1), which are most frequently reported among confined populations [26]. In our investigation, thiamine levels were within normal ranges, possibly due in part to a national policy of thiamine fortification of rice. While some copper deficiency was identified, it cannot explain disease in all cases. Zinc has been associated with peripheral neuropathy in animal studies and is the only element in which all cases are deficient. However, a limitation of our investigation was the fact that we did not test controls without neuropathy, so we cannot determine the association of micronutrient deficiencies with neuropathy. While evidence of nutrient interdependency has been established, it is yet to be fully understood [27]. A lack of dietary diversity was significantly associated with peripheral neuropathy among IDPs. The dose-response relationship between the number of micronutrient-rich foods in the diet and peripheral neuropathy also points to the importance of dietary diversity for this population. It is likely that the lack of dietary diversification, including the intake of fish that is high in copper and other essential micronutrients, contributed to the peripheral neuropathies. Our findings suggest a possible association between multiple micronutrient deficiencies or a newly identified association between zinc deficiency and peripheral neuropathy in humans.

In addition to infectious agents, peripheral neuropathy has also been associated with medications and toxicity, including arsenic, which can leech from volcanic rocks into aquifers and may be a threat to drinking-water sources of volcano-dwelling populations [28]. In our investigation, there was no evidence of arsenic or heavy metals in human or environmental samples, and none of the cases reported exposure to relevant medication.

This study has several limitations. The 90-day food history may have introduced the possibility of recall bias, however the monotony of the food rations provided and the lack of access to other foods mean the findings may be more valid than if used in other settings. While this population may have been more vulnerable to cholera, our assessment of the impact of micronutrient deficiencies on cholera mortality is limited by the paucity of data among fatal cases and the controls.

In summary, we described co-occurrence of cholera and peripheral neuropathy in a vulnerable, displaced population. The peripheral neuropathies were likely caused by complex micronutrient deficiencies linked to non-diversified diets that potentially increased the vulnerability of this population; however a new zinc associated neuropathy could not be ruled out. The co-occurrence of acute outbreak-related disease with underlying co-morbidity such as peripheral neuropathy in displaced populations highlights the complexity of the disease in the group and the need to address broader problems. Upsurges in violence, crop destruction and natural disasters can cause sharp increases in food insecurity, forcing the displacement of vulnerable populations that may experience high mortality rates during the period immediately following their migration [15]. Preventing the reoccurrence of such events in Papua New Guinea will be achieved by addressing the root cause of violence and insecurity: access to arable land and timely resettlement.
